# *Gas6* Downregulation Impaired Cytoplasmic Maturation and Pronuclear Formation Independent to the MPF Activity

**DOI:** 10.1371/journal.pone.0023304

**Published:** 2011-08-05

**Authors:** Kyeoung-Hwa Kim, Eun-Young Kim, Yuna Kim, Eunju Kim, Hyun-Seo Lee, Sook-Young Yoon, Kyung-Ah Lee

**Affiliations:** Department of Biomedical Science, College of Life Science, Fertility Center, CHA Research Institute, CHA University, CHA General Hospital, Seoul, Korea; Ottawa Hospital Research Institute and University of Ottawa, Canada

## Abstract

Previously, we found that the growth arrest-specific gene 6 (*Gas6*) is more highly expressed in germinal vesicle (GV) oocytes than in metaphase II (MII) oocytes using annealing control primer (ACP)-PCR technology. The current study was undertaken to investigate the role of *Gas6* in oocyte maturation and fertilization using RNA interference (RNAi). Interestingly, despite the specific and marked decrease in *Gas6* mRNA and protein expression in GVs after *Gas6* RNAi, nuclear maturation including spindle structures and chromosome segregation was not affected. The only discernible effect induced by *Gas6* RNAi was a change in maturation promoting factor (MPF) activity. After parthenogenetic activation, *Gas6* RNAi-treated oocytes at the MII stage had not developed further and arrested at MII (90.0%). After stimulation with Sr^2+^, *Gas6*-silenced MII oocytes had markedly reduced Ca^2+^ oscillation and exhibited no exocytosis of cortical granules. In these oocytes, sperm penetration occurred during fertilization but not pronucleus (PN) formation. By roscovitine and colcemid treatment, we found that the *Gas6* knockdown affected cytoplasmic maturation directly, independent to the changed MPF activity. These results strongly suggest that 1) the *Gas6* signaling itself is important to the cytoplasmic maturation, but not nuclear maturation, and 2) the decreased *Gas6* expression and decreased MPF activity separately or mutually influence sperm head decondensation and PN formation.

## Introduction

Mammalian oocytes in ovarian follicles have arrested growth and a large nucleus known as a germinal vesicle (GV). The meiotic cell cycle is arrested at the diplotene stage of the first prophase, and some selective oocytes initiate growth following gonadotropin simulation [1], [2]. Oocyte growth and maturation are long and requisite processes for fertilization and subsequent embryo development until embryonic genome activation starts. Oocyte maturation involves nuclear and cytoplasmic maturation. Although strictly linked, these are complex and different events [3], [4], [5].

The process of nuclear maturation, meiotic cell cycle, involves GV breakdown (GVBD), chromosome condensation and segregation, organization of microtubules, and release of the first polar body, after which oocytes are arrested again at metaphase II (MII) until fertilization [3]. This process is mainly controlled by a phosphorylation and/or dephosphorylation regulatory cascade of maturation promoting factor (MPF) and mitogen-activated protein kinase (MAPK) [6], [7], [8].

The process of cytoplasmic maturation involves organelle reorganization, cytoskeleton dynamics and molecular maturation during oocyte growth and meiosis [9]. Organelles such as mitochondria, ribosomes, endoplasmic reticulum, cortical granules, and the Golgi complex redistribute to the cytoplasm during oocyte maturation. Cytoskeletal microfilaments and microtubules function in spindle formation and chromosome segregation.

Oocytes accumulate maternal mRNA, protein, and regulatory molecules that function in the completion of meiosis, fertilization, and early embryogenesis [10]. This process is mainly controlled by post-transcriptional regulatory mechanisms, such as RNA polyadenylation, localization, sorting, and masking, as well as protein phosphorylation [11]. Therefore, functional analysis of certain gene(s) in the oocyte should provide important information on the molecular regulatory mechanism of oocyte nuclear and cytoplasmic maturation, fertilization, and early embryogenesis [12], [13], [14].

Oocytes underwent nuclear and cytoplasmic maturation, resulting in arrest at meiotic MII. Sperm penetration breaks this arrest and requires recognition of the zona pellucida (ZP), dependent upon three ZP proteins (ZP1-3) [15]. Sperm undergo the acrosome reaction and penetrate the ZP [16]. Sperm bind the ooplasma through interactions with microvilli and associated membrane proteins and ultimately form a fusion pore [17]. The oscillatory Ca^2+^ signal is necessary and sufficient for the resumption of meiosis and cortical granule release, resulting in the blockade of polyspermy and extrusion of the second polar body [18], [19]. To complete the fertilization process, pronuclei (PNs) are formed through the remodeling of paternal and maternal chromatin. Subsequently, maternal and fraternal PNs migrate for the preparation of syngamy [20], [21], [22], [23].

In a previous study, it was found that growth arrest-specific gene 6 (*Gas6*) is highly expressed in GV oocytes by using annealing control primer (ACP)-polymerase chain reaction (PCR) [24]. *Gas6* is a member of the vitamin K-dependent protein family and a ligand for *Axl*, *Tyro3*, and *Mertk* receptor tyrosine kinases [25], [26]. It has been reported that *Gas6*-mediated signaling is implicated in cell survival, growth arrest, proliferation, differentiation, and other cell type-specific functions [26], [27], [28], [29]. Clinically, *Gas6* plays an important role in hematosis and thrombosis [26]. At present, the function of *Gas6* and receptor signaling has been studied in thrombosis and spermatogenesis, but not in oocytes and embryos [26], [30]. Thus, the objective of the present study was to evaluate the roles of *Gas6* in oocytes, completion of the meiotic cell cycle, and fertilization and embryo development.

## Results

### *Gas6* mRNA expression in oocytes and embryos

#### During oocyte maturation

**Figure 1:**
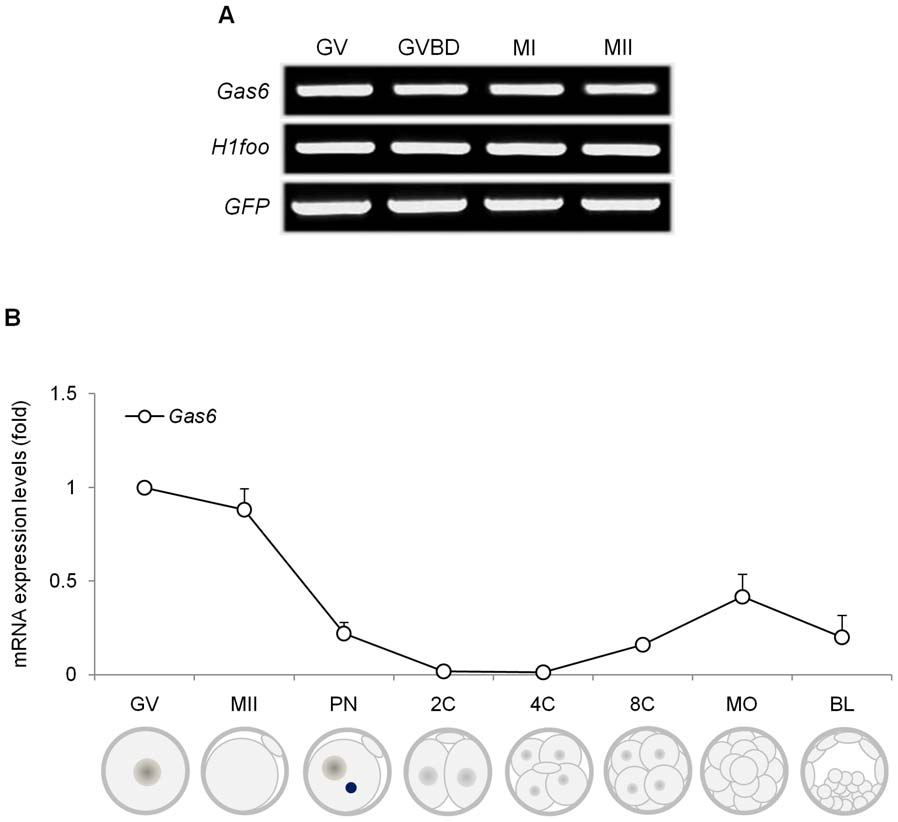
Differential expression of *Gas6* during oocyte maturation and early embryogenesis. (**A**) Typical pattern of *Gas6* expression during oocyte maturation. The mRNA equivalent to a single oocyte taken after culture for 0, 2, 8, and 16 hours, corresponding to GV, GVBD, MI, and MII stages, respectively, was used for each lane. *H1foo* used as an internal control. *GFP* was used as an external control to measure equal recovery. (**B**) Expression of *Gas6* during early embryogenesis. Relative gene expression of *Gas6* in a single oocyte and single embryo throughout the developmental stages was measured by quantitative real-time PCR. Relative expression levels of *Gas6* were calculated from C_T_ values and normalized to added *GFP* synthetic RNA, and the expression ratio was calculated against *Gas6* expression in the GV oocyte. Experiments were repeated at least three times, and data were expressed as mean ± SEM.

During oocyte maturation, the level of polyadenylated mRNA is reduced by more than 50% through deadenylation [31], [32]. Previously, it was found that the mRNA expression of certain genes required for oocyte maturation or embryogenesis, namely *Sebox* and *Obox4*, was markedly decreased during oocyte maturation [12], [33]. However, the expression of *Gas6* was constitutive throughout oocyte maturation (Fig. 1A). Therefore, the abundant *Gas6* mRNA in the GV stage is not deadenylated during meiotic maturation and is maintained as polyadenylated mRNA in the MII stage.

#### During embryogenesis

The expression of *Gas6* during preimplantational embryo development was evaluated by quantitative real-time PCR. In each reaction, the absence of nonspecific amplification was confirmed by checking the melting curve of each gene with a single peak. The *Gas6* transcript was highly expressed in GV and MII oocytes, gradually decreased to an undetectable level from PN to 2-cell (2C) and 4-cell (4C) stage embryos, and slightly increased again from 8-cell (8C) to blastocyst stage embryos (Fig. 1B).

### *Gas6* RNAi had no effect on oocyte nuclear maturation but effect on oocyte cytoplasmic maturation

RNAi by microinjection of double-stranded RNA (dsRNA) is a suitable tool for studying gene function in the oocytes and embryos of mammalian species [34], [35], [36]. In the present study, we demonstrated that microinjection of *Gas6* dsRNA into the cytoplasm of GV oocytes led to selective interference of maternal mRNA and subsequent protein expression and that *Gas6* RNAi inhibited the cytoplasmic maturation of oocytes and fertilization.

#### Oocyte maturation after *Gas6* RNAi

**Figure 2 pone-0023304-g002:**
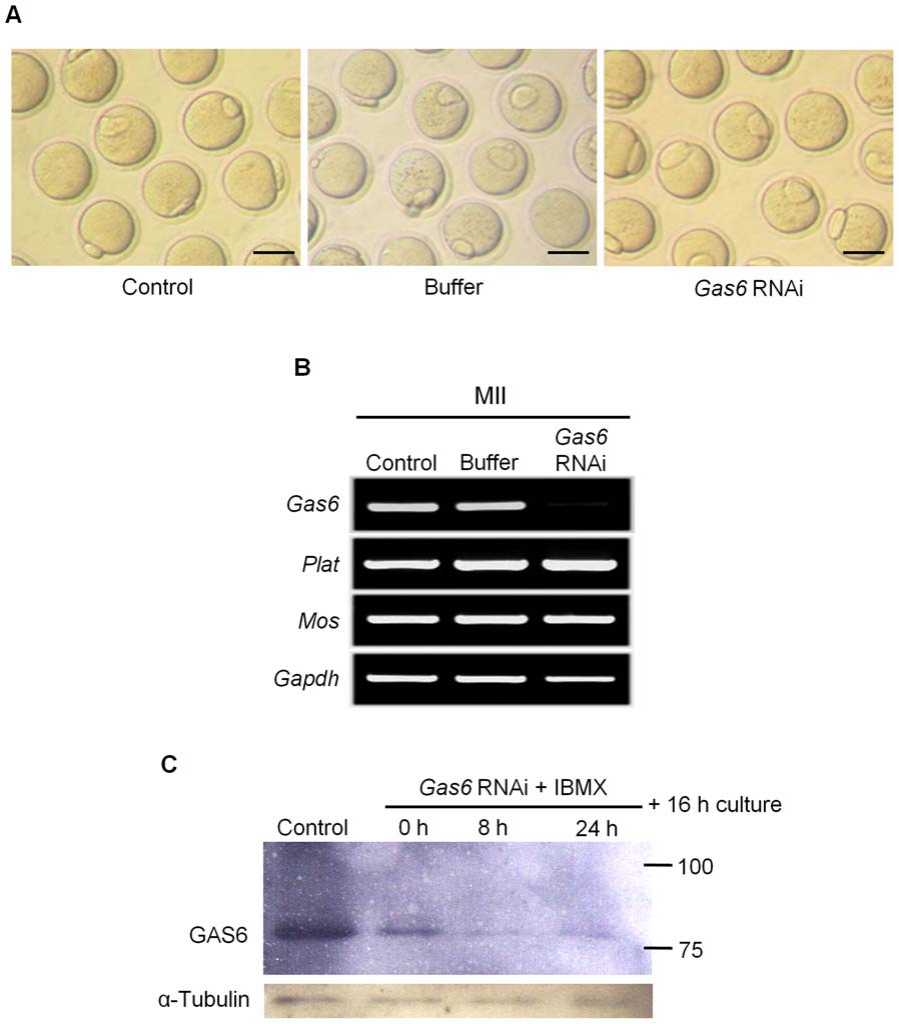
*Gas6*-silenced oocytes matured normally to the MII stage. (**A**) Micrographs of MII oocytes without or with *Gas6* RNAi. Scale bars indicate 100 µm. (**B**) Typical gene expression profiles for targeted and untargeted genes (*Plat*, *Mos*, and *Gapdh*) in *Gas6*-silenced MII oocytes were determined using RT-PCR analysis. *Gas6* RNAi treatment resulted in the specific suppression of *Gas6* expression. (**C**) Western blot analysis of GAS6 expression after *Gas6* RNAi treatment demonstrated the complete knockdown of GAS6 protein expression. The numbers 100 and 75 (kDa) on the right side of the figure are the size markers of the protein. Protein lysates of 500 MII oocytes were loaded per lane. α-Tubulin was used as a loading control. Control, control MII oocytes cultured for 16 hours in medium alone; 0 h, *Gas6* dsRNA-injected oocytes cultured for 16 hours in plain medium alone; 8 h, *Gas6* dsRNA-injected oocytes cultured in IBMX-supplemented medium for 8 hours followed by 16 hours in plain medium; 24 h, *Gas6* dsRNA-injected oocytes cultured in IBMX-supplemented medium for 24 hours followed by 16 hours in plain medium.

After treatment with *Gas6* RNAi, oocytes matured with similar MII rates (*Gas6* RNAi; 81.9%) as those of the control (86.9%) and buffer-injected (78.4%) groups (Table 1). The MII oocytes that developed from GV oocytes injected with *Gas6* dsRNA were morphologically normal compared to the control oocytes (Fig. 2A) despite the specific and marked depletion of *Gas6* mRNA (Fig. 2B). To verify the sequence-specific knockdown of the target RNA, we measured the expression levels of targeted and untargeted genes and found that the expression of untargeted genes, such as *Plat*, *Mos*, and *Gapdh*, remained unchanged in *Gas6*-targeted MII oocytes despite the decrease in *Gas6* mRNA expression. These results confirmed that *Gas6* RNAi caused sequence-specific *Gas6* knockdown (Fig. 2B).

**Table 1:** In vitro maturation of mouse oocytes after injection of *Gas6* dsRNA into GV oocytes.

	Number of oocytes (%)			
	Total	Germinal vesicle (GV)	Metaphase I (MI)	Metaphase II (MII)
Control	168	0 (0) ^a^	22 (13.1)	164 (86.9) ^a^
Buffer	166	20 (12) ^b^	16 (9.6)	130 (78.4) ^b^
*Gas6* RNAi	215	7 (3.2) ^ac^	32 (14.9)	176 (81.9) ^ab^

a, b, c Different letters indicate significant difference at *p<*0.05.

To confirm the complete knockdown of GAS6 protein expression, we performed Western blot analysis for GAS6 protein after treatment with *Gas6* RNAi followed by 8 or 24 hours of in vitro culture of oocytes in 3-isobutyl-1-methyl-xanthine (IBMX) medium and in vitro maturation for 16 hours. High levels of endogenous GAS6 expression were detected in control MII oocytes that had matured in vitro for 16 hours (Fig. 2C, Lane 1). We found that GAS6 expression decreased after treatment with *Gas6* RNAi (Fig. 2C, Lanes 2–4) and almost completely disappeared in MII oocytes following 8 or 24 hours of preincubation in IBMX (Fig. 2C, Lanes 3, 4). These data suggest that the complete knockdown of GAS6 protein occurred after 8 hours of preincubation in IBMX-supplemented medium.

#### Spindle and chromosomal configuration

**Figure 3 pone-0023304-g003:**
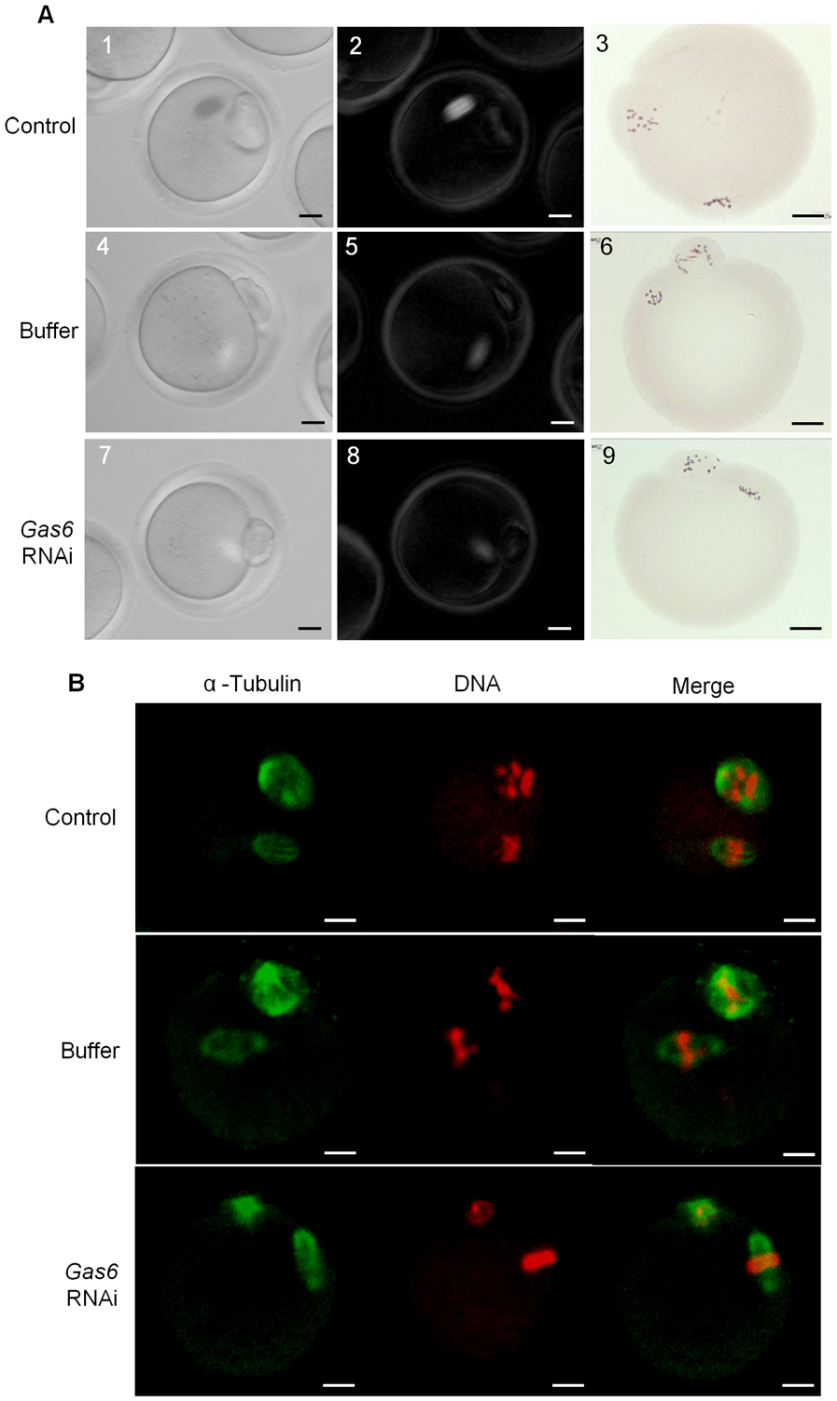
*Gas6* is not required for spindle and chromosomal dynamics in meiotic cell cycle. (**A**) Microphotographs in the left panel (1, 4, 7) show the oocytes under bright field, whereas microphotographs in the middle panel (2, 5, 8) show the noninvasive analysis of spindle structure in same oocytes using Polscope. Microphotographs in the right panel (3, 6, 9) show the results of aceto-orcein staining of chromosomes in MII oocytes. (**B**) Immunofluorescence staining for spindle and chromosome morphology in control and *Gas6* dsRNA-injected oocytes matured in vitro. MII oocytes were fixed in 4% paraformaldehyde, stained with α-tubulin antibody (Green), and counterstained with propidium iodide (Red) for DNA staining. Scale bars indicate 25 µm.

There was no change in the shape and localization of meiotic spindles and chromosomes in MII oocytes after *Gas6* knockdown (Fig. 3A, 1–9). The normal shape and size of the meiotic spindle and chromosomal alignments were additionally confirmed by immunofluorescence staining (Fig. 3B). Taken together, these results suggest that *Gas6* is not involved in the resumption, progression, and completion of the normal meiotic cell cycle.

#### Decrease of MPF activity after *Gas6* RNAi

**Figure 4 pone-0023304-g004:**
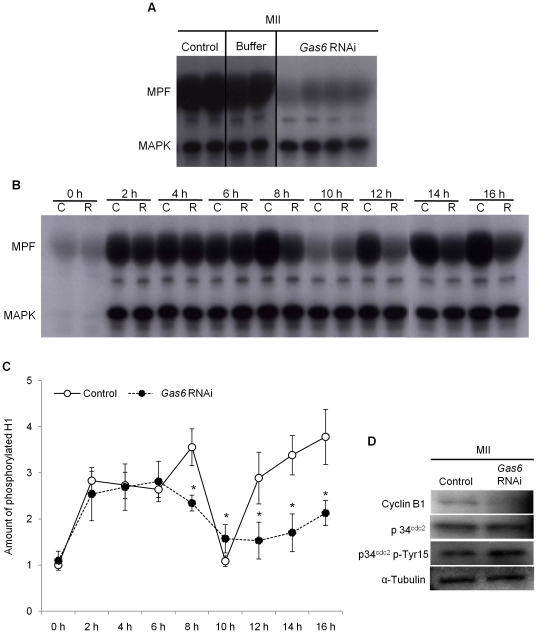
*Gas6* RNAi impaired reactivation of MPF after emission of the first polar body. (**A**) To assess the activities of MPF and MAPK after *Gas6* RNAi, phosphorylation of the substrates histone H1 and MBP, reflecting the kinase activities of MPF and MAPK, respectively, was determined. Each lane contained one MII stage oocyte. Control, uninjected oocytes; Buffer, buffer-injected for sham control oocytes; *Gas6* RNAi, *Gas6* dsRNA-injected oocytes. (**B**) Dual kinase activity analysis to determine the phosphorylation levels of the substrates histone H1 and MBP was performed every 2 hours after *Gas6* RNAi injection. C, uninjected control oocytes; R, *Gas6*-depleted oocytes. (**C**) The amount of phosphorylated histone H1 was calculated, and relative amounts were presented in line graphs. Asterisks represent statistical significance at *p<*0.05. Intact line with open circles, Control, uninjected oocytes; Dotted line with closed circles, *Gas6* RNAi, *Gas6*-silenced oocytes. (**D**) Western blot analysis of cyclin B1, p34^cdc2^, and p34^cdc2^ p-Tyr15 in MII oocytes after *Gas6* RNAi treatment. Depletion of GAS6 protein after *Gas6* RNAi treatment affected MPF activity. Protein lysates of 250 MII oocytes were loaded per lane. α-Tubulin was used as a loading control. Control, uninjected control MII oocytes; *Gas6* RNAi, *Gas6*-silenced MII oocytes.

Mouse oocyte maturation is regulated by changes in MPF and MAPK activities [37], [38]. Therefore, we conducted a dual kinase activity assay to determine the effects of *Gas6* RNAi on the activities of MPF and MAPK. The activity of MPF in MII oocytes was dramatically decreased by *Gas6* RNAi treatment compared to the activity in the control group, whereas the activity of MAPK in MII oocytes was maintained at a level similar to that of the controls (Fig. 4A).

After confirming that *Gas6* RNAi had inhibitory effects on MPF activity, we determined the time course of inhibition by assaying activity every 2 hours after *Gas6* RNAi injection (Fig. 4B). In contol oocytes, MPF activity was increased after 2 hours, markedly decreased after 8–10 hours around the first polar body extrusion, and increased again and maintained through the MII stage. The level of MPF activity, therefore, closely corresponds to the nuclear events. In contrast, MPF activity in *Gas6*-depleted MII oocytes decreased 6–10 hours after in vitro maturation and did not increase again (Fig. 4C). These results suggest that *Gas6* is important in the reactivation of MPF after the first polar body emission but is not essential for the progression of nuclear maturation in mouse oocytes.

Cyclin B1 and p34^cdc2^ are essential components of active MPF. MPF activity is regulated by a translation-dependent mechanism that determines the level of cyclin B1 [39], [40]. To determine whether the inactivation of MPF in *Gas6*-depleted MII oocytes is cyclin B1-dependent, we measured cyclinB1-p34^cdc2^ expression by Western blotting. Interestingly, as shown in Fig. 4D, the expression of cyclin B1 was markedly decreased in *Gas6* dsRNA-injected oocytes relative to its expression in control oocytes. These results depict that *Gas6* RNAi caused MPF inactivation through cyclin B1 degradation. Moreover, although p34^cdc2^ expression was unchanged, p34^cdc2^ phospho-Tyr15 was upregulated in *Gas6*-depleted MII oocytes (Fig. 4D). These findings suggest that *Gas6* RNAi increased the phosphorylation of Tyr15 in p34^cdc2^, which resulted in MPF inactivation.

#### Parthenogenetic development after *Gas6* RNAi

**Figure 5 pone-0023304-g005:**
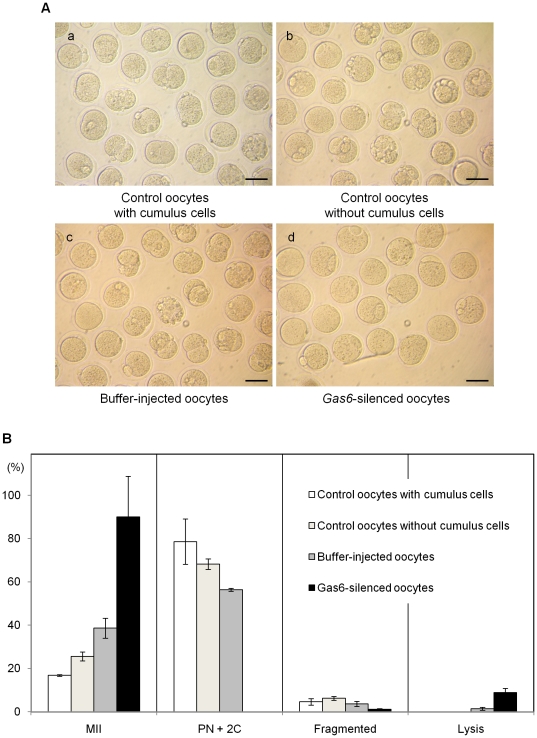
*Gas6*-silenced MII oocytes were not activated parthenogenetically. (**A**) Microphotographs of embryos after parthenogenetic activation. MII oocytes of various groups were activated by SrCl_2_ and cultured in CZB medium to observe parthenogenetic development. (a–c) Control oocytes developed to PN and 2C stages, whereas (d) *Gas6*-silenced oocytes were arrested at the MII stage. Scale bars indicate 100 µm. (**B**) Developmental stages of parthenogenetically activated oocytes. *Gas6*-silenced MII oocytes were all arrested at the MII stage and not activated.

When cytoplasmic maturation is not completed, oocytes fail to undergo fertilization and early embryo development [41], [42]. To confirm that normal cytoplasmic maturation was completed, parthenogenetic activation was performed using Sr^2+^ to stimulate the MII oocytes after *Gas6* RNAi treatment. Parthenogenetic activation in three control groups resulted in development to PN and 2C stages (Table 2, Fig. 5A,B). Following parthenogenetic activation, control oocytes with cumulus (Fig. 5Aa; 18.3% and 60.3%), control oocytes without cumulus (Fig. 5Ab; 28.7% and 39.5%), and buffer-injected sham control oocytes (Fig. 5Ac; 25.7% and 30.7%) developed to PN and 2C stages. However, 90% of the MII oocytes treated with *Gas6* RNAi (closed black bar in Fig. 5B) were not activated and were arrested at the MII stage (Fig. 5Atd), suggesting that *Gas6* plays a critical role in the initiation of cell cycle progression for early embryo development.

**Table 2:** Development after parthenogenetic activation by Sr^2+^ stimulation.

	Number of activated oocytes (%)					
	Total	MII	PN	Two cells	Fragmented	Lysis
Control oocyteswith cumulus cells	131	22 (16.8) ^a^	24 (18.3) ^a^	79 (60.3) ^a^	6 (4.9)	0 (0) ^a^
Control oocytes without cumulus cells	129	33 (25.6) ^ab^	37 (28.7) ^b^	51 (39.5) ^ab^	8 (6.2)	0 (0) ^a^
Buffer-injected oocytes	140	54 (38.6) ^bc^	36 (25.7) ^ab^	43 (30.7) ^b^	5 (3.6)	2 (1.4) ^ab^
*Gas6*-silenced oocytes	90	81 (90) ^d^	0 (0) ^c^	0 (0) ^c^	1 (1.1)	8 (8.9) ^bc^

a, b, c, d Different letters indicate significant difference at *p<*0.05.

### Effects of *Gas6* RNAi on fertilization and PN formation

Based on these results, we hypothesized that the reduction of *Gas6* expression may have resulted in fertilization failure. Therefore, we conducted in vitro fertilization and evaluated the changes in Ca^2+^ oscillation in MII oocytes and the rates of sperm penetration and PN formation. For measuring the exocytosis of cortical granules, fluorescein isothiocyanate (FITC)-*Lens culinaris* agglutinin (LCA) staining was performed following Sr^2+^-induced activation.

#### Sperm penetration but no PN formation after *Gas6* RNAi

**Figure 6 pone-0023304-g006:**
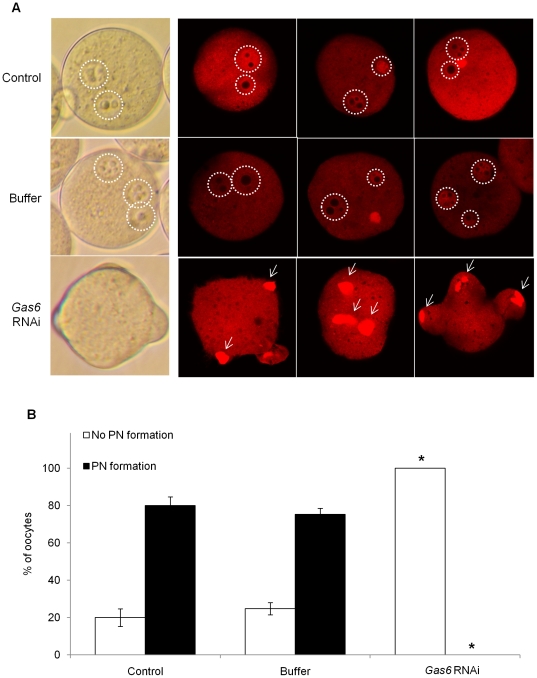
Sperm penetration, but not pronuclear formation, occurred in *Gas6*-silenced MII oocytes. (**A**) After in vitro fertilization, eggs were fixed in 4% paraformaldehyde and stained with propidium iodide (red) to confirm the sperm DNA. Control eggs had both maternal and paternal PN formation through chromatin remodeling. On the contrary, *Gas6*-silenced eggs exhibited sperm penetration but not fully decondensed paternal chromatin in the cytoplasm. PN formation was not observed. White dot circles indicate PN formation, and white arrows indicate not fully decondensed paternal chromatin before PN formation. Control, uninjected eggs; Buffer, buffer-injected for sham control eggs; *Gas6* RNAi, *Gas6-*silenced eggs. (**B**) Percentage of oocytes with PN formation after in vitro fertilization. The black columns indicate the percentage of oocytes with PN formation, and the white columns indicate the percentage of oocytes without PN formation.

Concurrent with activation, sperm nuclear contents and paternal chromatin undergo biochemical remodeling via resources within the cytoplasm of oocytes [20]. Due to insufficient cytoplasmic maturation, oocytes failed to undergo fertilization. After several failures of in vitro fertilization with ZP-intact oocytes, we performed in vitro fertilization with ZP-free MII oocytes and monitored sperm penetration and PN formation. Following in vitro fertilization, uninjected and buffer-injected control oocytes exhibited high rates (>70%) of PN formation after sperm penetration (Fig. 6A, B). Because we used ZP-free oocytes, we could observe two or three PNs. In contrast, the majority of the eggs treated with *Gas6* RNAi failed to undergo the remodeling of paternal and maternal chromatin and PN formation despite sperm penetration into the cytoplasm (Fig. 6A, B). We confirmed that sperm penetration had occurred in *Gas6* RNAi-treated MII oocytes by finding partially decondensed paternal chromosomes in the cytoplasm (Fig. 6A, arrows). These results indicate that *Gas6*-silenced MII oocytes had insufficient cytoplasmic maturation that resulted in the failure of PN formation.

### Effects of *Gas6* RNAi on insufficient cytoplamic maturation

#### Calcium oscillations in *Gas6*-silenced MII oocytes

**Figure 7 pone-0023304-g007:**
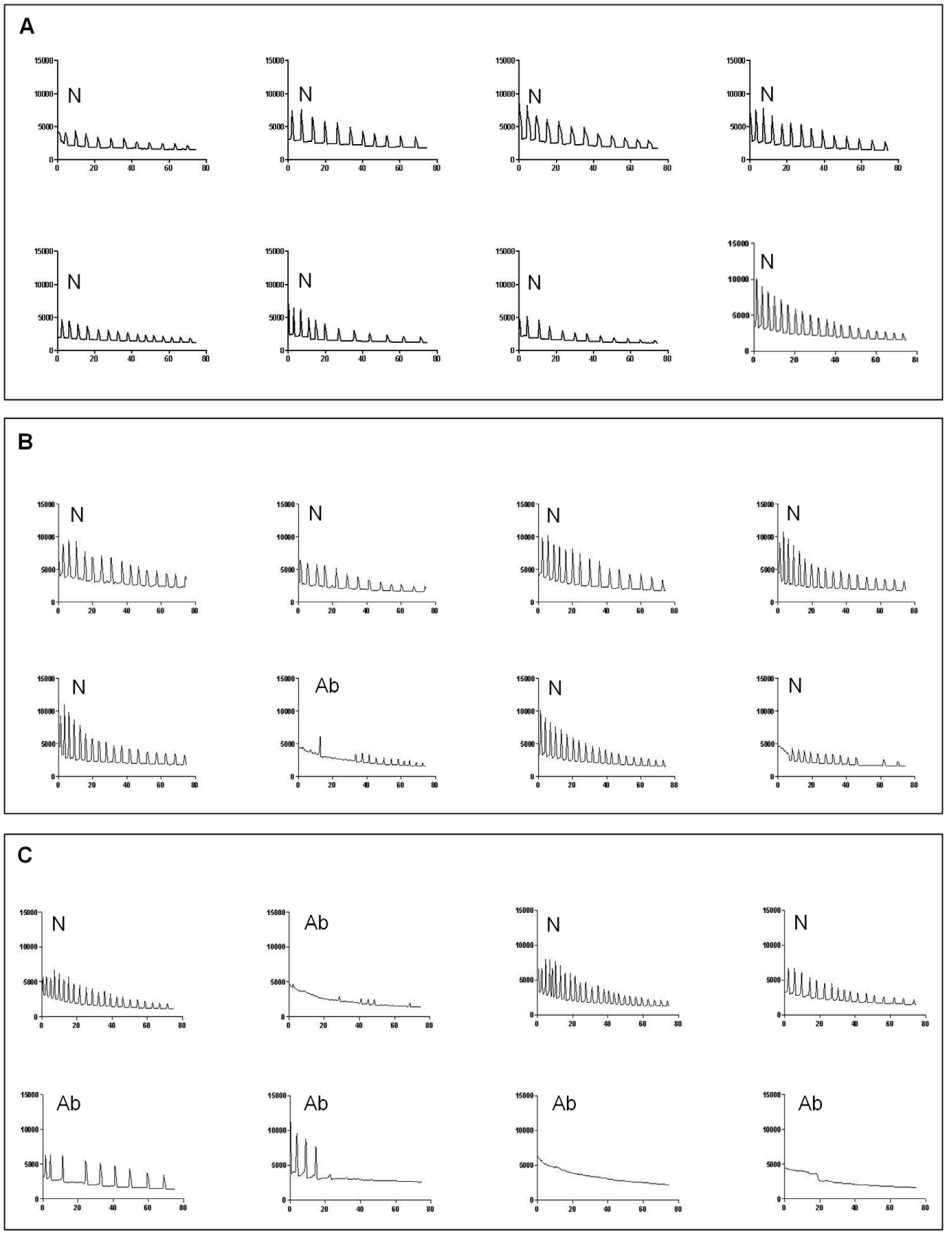
Representative Ca^2+^ oscillatory patterns in Sr^2+^-induced MII oocytes. Changes in the fluorescence of a Ca^2+^ indicator dye in mouse oocytes followed by incubation in Sr^2+^-containing medium revealed the almost normal pattern of Ca^2+^ oscillations in the control (**A**) and buffer-injected sham control (**B**) MII oocytes. However, *Gas6*-silence MII oocytes (**C**) exhibited both normal and abnormal patterns of Ca^2+^ oscillations. N, normal Ca^2+^ oscillatory pattern; Ab, abnormal Ca^2+^ oscillatory pattern.

During fertilization, sperm break the arrest of the second meiotic cycle of the oocytes by inducing a series of repetitive Ca^2+^ oscillations that persist for several hours and terminate upon PN formation [43], [44]. This event has been confirmed in many rodents and in human oocytes [45]. To determine if a reduction in *Gas6* expression affects the Ca^2+^ oscillations in MII oocytes, MII oocytes were stimulated with SrCl_2_. When the Ca^2+^ oscillation patterns in each group were analyzed (Table 3), most of the uninjected and buffer-injected oocytes exhibited normal patterns of oscillatory Ca^2+^ activity (Fig. 7A, B). In *Gas6*-scilenced oocytes, the frequency of the abnormal pattern of Ca^2+^ oscillation increased (65.4%, Fig. 7C).

**Table 3:** Intracellular Ca^2+^ oscillation after Sr^2+^ stimulation in *Gas6*-silenced MII oocytes.

	Number of Sr^2+^-induced MII oocytes (%)		
	Total	With normal Ca^2+^ spikes	With abnormal Ca^2+^ spikes
Control	20	15 (75.0) ^a^	25 (25.0) ^a^
Buffer	22	17 (77.3) ^a^	5 (22.7) ^a^
*Gas6* RNAi	26	9 (34.6) ^b^	17 (65.4) ^b^

a, b Different letters indicate significant difference at *p<*0.05.

#### Cortical granule exocytosis in *Gas6*-silenced MII oocytes

**Figure 8 pone-0023304-g008:**
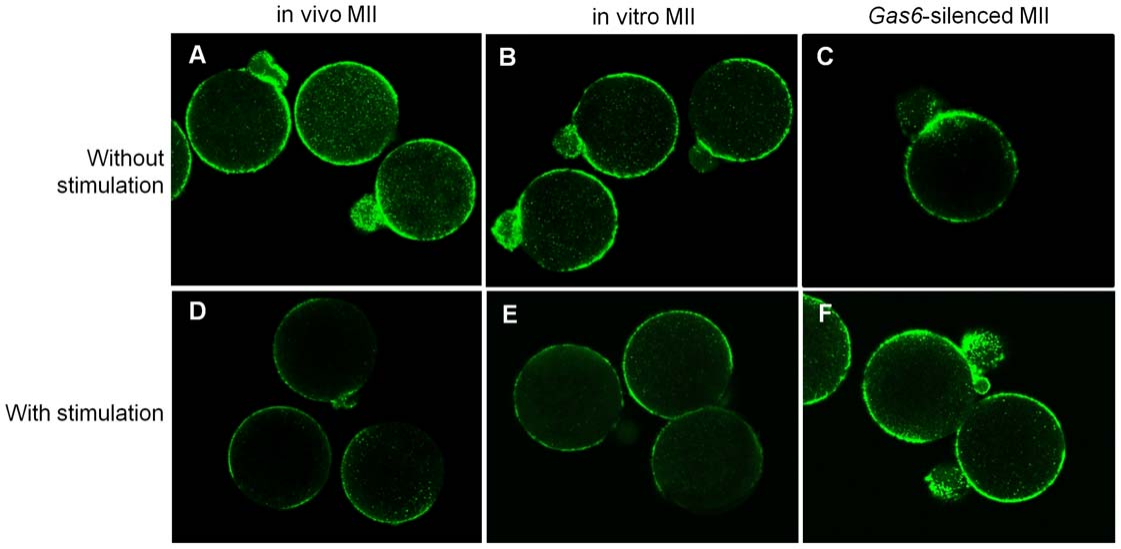
Abnormality in cortical granule exocytosis was induced in *Gas6*-silenced MII oocytes. Uninjected MII (in vivo and in vitro) and *Gas6*-silenced MII oocytes cultured for 3 hours with or without 10 mM SrCl_2_ (with stimulation and without stimulation, respectively). Sr^2+^-treated uninjected MII oocytes exhibited the release of cortical granules (D, E). However, cortical granule exocytosis was inhibited in *Gas6*-silenced MII oocytes (F). Green fluorescence indicates FITC-labeled cortical granules.

The normal Ca^2+^ oscillation in eggs is essential for cortical granule release. In the present study, *Gas6* RNAi caused low cytoplasmic Ca^2+^ excitability. We further examined cortical granule exocytosis with SrCl_2_ stimulation. Without SrCl_2_ treatment, MII oocytes in all three groups exhibited the existence of intact cortical granules (green fluorescence) around the cortices of oocytes (Fig. 8A–C). Upon treatment, SrCl_2_-treated in vivo and in vitro MII oocytes exhibited decreased green fluorescence around their cortices, indicating that cortical granule exocytosis had occurred (Fig. 8D, E). However, most of the *Gas6-*silenced MII oocytes exhibited intact green fluorescence, indicating unsuccessful cortical granule exocytosis in these oocytes (Fig. 8F). These results imply that decreased cytoplasmic Ca^2+^ excitability after *Gas6* RNAi prevented cortical granule release and caused fertilization failure.

#### *Gas6* decrease directly affected failure of fertilization

**Figure 9:**
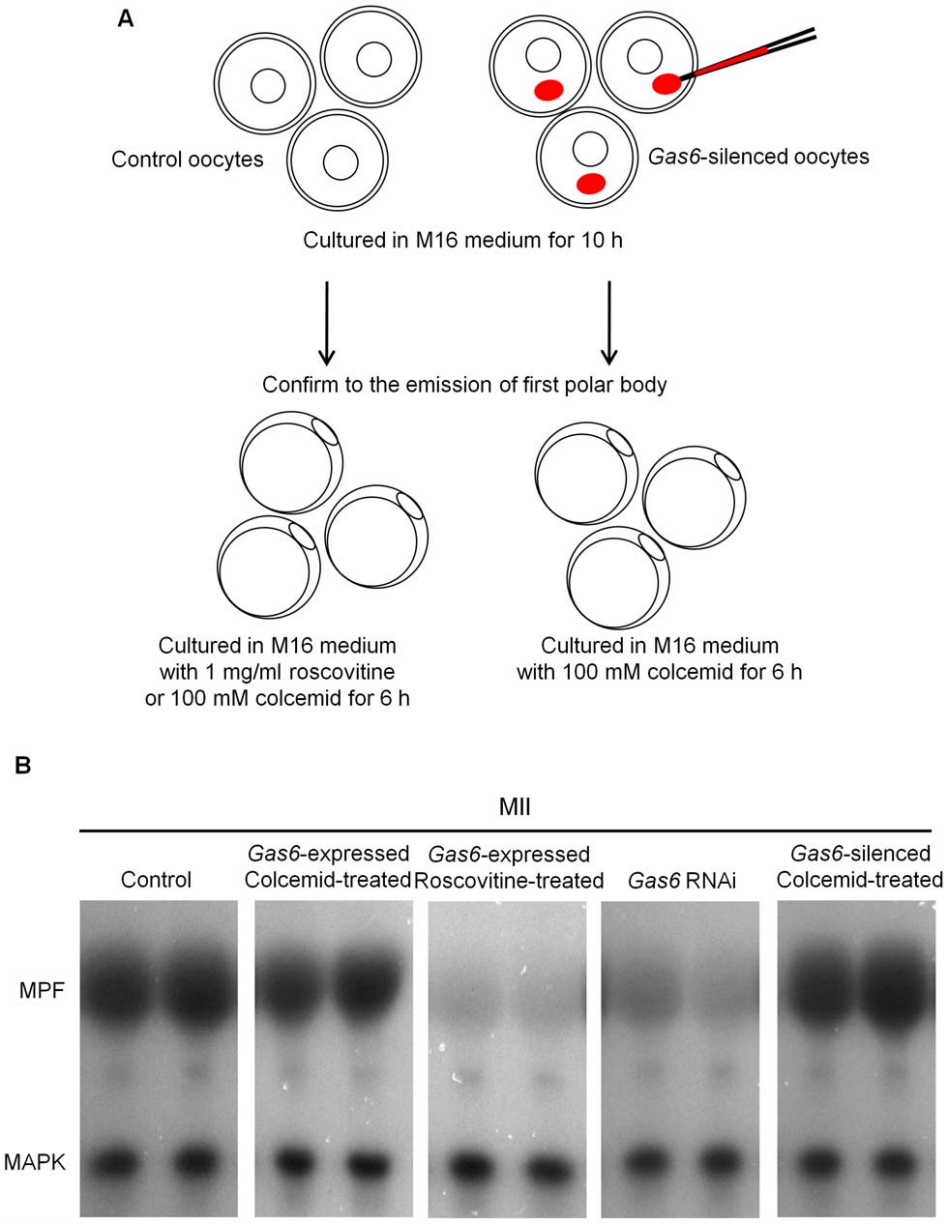
Roscovitine and colcemid treatment to regulate the MPF activity in MII oocytes. (**A**) Schematic outline of the experimental procedure. Roscovitine, a specific inhibitor of p34^cdc2^-cyclin B1 kinase, was used to inactivate MPF. Control oocytes were cultured in M16 medium for 10 hours, and then transferred in M16 medium containing roscovitine, when first polar body was emitted. Colcemid, a drug for preventing the degradation of Cyclin B, was used to maintain MPF activity. Control and *Gas6*-dsRNA injected oocytes were cultured in M16 medium for 10 hours until the first polar body extrusion followed by culture in M16 medium containing colcemid for 6 hours. (**B**) Dual kinase activity analysis to determine the activation levels of the MPF and MAPK was performed. Roscovitine-treated oocytes suppressed activity of MPF and colcemid-treated oocytes maintained activity of MPF.

**Figure 10:**
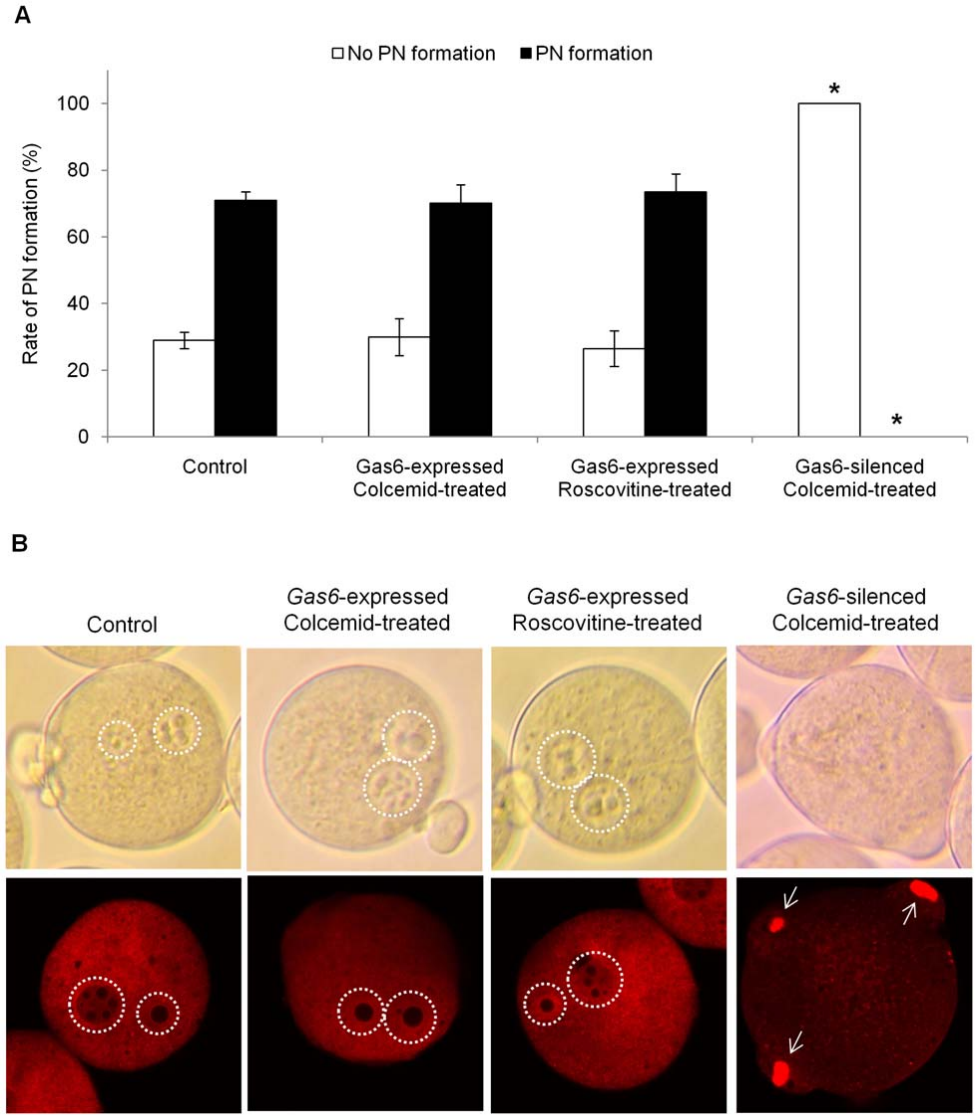
Decrease of *Gas6* expression independent to the MPF activity resulted in failure of PN formation. (**A**) Percentage of oocytes with PN formation after in vitro fertilization. The black columns indicate the percentage of oocytes with PN formation, whereas the white columns indicate the percentage of oocytes without PN formation. (**B**) Control and *Gas6*-expressed colcemid or roscovitine treatment eggs had PN formation, while colcemid-treated *Gas6*-silenced eggs exhibited no PN formation. After in vitro fertilization, eggs were fixed in 4% paraformaldehyde and stained with propidium iodide (red). White dot circles indicate PN formation, and white arrows indicate not fully decondensed paternal chromatin. Control, uninjected oocytes; *Gas6*-expressed colcemid-treated, control oocytes cultured in M16 medium for 10 hours followed by 6 hours of culture in colcemid containing medium; *Gas6*-expressed rocovitine-treated, control oocytes cultured in M16 medium for 10 hours followed by 6 hours of culture in roscovitine containing medium; *Gas6*-silenced colcemid-treated, *Gas6* dsRNA-injected oocytes cultured in M16 medium for 10 hours followed by 6 hours of culture in colcemid containing medium.

To address whether the depletion of MPF activity due to reduction of *Gas6* expression is indeed responsible for failure of fertilization, *Gas6*-expressed control and *Gas6*-silenced oocytes were cultured with roscovitine (inhibit MPF activity) or colcemid (maintain MPF activity) after first polar body extrusion, as explained in the diagram Fig. 9A. We confirmed that the activity of MPF in *Gas6*-expressed MII oocytes with roscovitine treatment was markedly reduced, while the activity of MPF in colcemid-treated MII oocytes after *Gas6* RNAi was rescued at a similar level to that of the control oocytes (Fig. 9B). When the oocytes were fertilized at this time point, 71.0% of control and 70.1% of *Gas6*-expressed, high MPF activity oocytes with colcemid treatment had PN formation. Also, 73.5% of *Gas6*-expressed, low MPF activity oocytes with roscovitine treatment had PN formation, but none of *Gas6*-sileced, high MPF activity oocytes with colcemid treatment had PN formation (Fig. 10A, B). Therefore, these results undoubtedly indicate that neither low MPF activity nor colcemid effect, but *Gas6* knockdown resulted in the insufficient cytoplasm maturation and consequently the failure of PN formation.

## Discussion

In this study, we investigated the expression of *Gas6* in mouse oocytes and embryos, and demonstrated that *Gas6* RNAi caused the disturbance of oocyte cytoplasmic maturation with a concurrent failure of fertilization. These results strongly suggest that *Gas6* is a new candidate mammalian maternal effect gene.

Translation in oocytes is controlled by cytoplasmic polyadenylation and deadenylation of maternal transcripts, which are associated with the addition or loss of adenosine residues on the 3′-untranslated region of mRNA [46]. We observed that polyadenylated *Gas6* mRNA is highly expressed in MII oocytes, and this constitutively abundant *Gas6* mRNA throughout oocyte maturation may play an essential role in oocyte cytoplasmic maturation and is directly related to early embryo development until zygotic gene activation starts. Previously, we observed that the mRNA expression of *Bcl2l10* is also highly expressed in MII oocytes compared with its expression in GV oocytes, but *Bcl2l10* RNAi treatment resulted in metaphase I (MI) arrest [14]. At this time, however, we cannot explain why *Bcl2l10* RNAi resulted in MI arrest whereas *Gas6* RNAi resulted in MII arrest.

Studies in mammalian oocytes revealed that the dynamic events of polymerization of microtubules and microfilaments are crucial in spindle formation, chromosome segregation, and extrusion of the polar body during oocyte meiotic maturation [39], [47]. In the present study, despite the significant reduction of the transcript and protein content of *Gas6*, oocytes developed to a morphologically normal MII stage with the first polar body. Additionally, we observed completion of meiosis with normal meiotic spindle organization and chromosome arrangement. Consequently, we concluded that *Gas6* is not involved in oocyte meiotic maturation, especially in the dynamics of microtubules and microfilaments.

MPF, a key regulator of both mitotic and meiotic cell cycles, consists of a catalytic subunit (p34^cdc2^) and a regulatory subunit (cyclin B1) [7]. Cyclin B1 plays an important role in regulating MPF action. Degradation of cyclin B1 and dissociation of the p34^cdc2^-cyclin B1 complex without cyclin B1 proteolysis leads to MPF inactivation. It also has been reported that phosphorylation of Thr14 and Tyr15 in p34^cdc^2 inhibits MPF activity [48]. In the present study, *Gas6* knockdown in MII oocytes led to cyclin B1 degradation and subsequent MPF inactivation. Moreover, we observed that *Gas6*-silenced MII oocytes had increased phosphorylation of Tyr15 in p34^cdc2^. Therefore, we concluded that the decrease in cyclin B1 levels and increased phosphorylation of Tyr15 in p34^cdc2^ reduced MPF activity in *Gas6*-silenced MII oocytes.

When MPF activation was inhibited in oocytes, meiotic resumption was prevented, and oocytes arrested at the GV stage [49]. Blocking MPF activity in oocytes before or immediately after the first polar body extrusion also prevented MII entry and led the oocytes into interphase, as manifested by the presence of a well-defined nucleus and decondensed chromosome. These findings suggest that MPF reactivation is absolutely necessary for preventing oocyte entry into interkinesis and the MII stage [50]. Surprisingly, however, *Gas6*-silenced oocytes with decreased MPF activity progressed into the MII stage with morphological demonstration of the first polar body emission. That is because the activity of MPF of the *Gas6*-silenced MII was reduced after extrusion of the first polar body; thus, it did not affect the nuclear maturation of oocytes.

After polar body emission, cyclin B degradation stops, and the anaphase promoting complex/cyclosome is reactivated upon entry into the second meiotic metaphase by high levels of MPF. The equilibrium between the slow degradation and the continuous synthesis of cyclin B depends on cytostatic factor (CSF), and MPF activity is maintained at high levels by CSF in MII oocytes [39], [51]. The cell cycle progression of unfertilized oocytes is arrested at the MII stage until fertilization or activation occurs [52]. The maintenance of MII arrest requires CSF activity through a Mos/MEK/MAPK/p90^rsk^-dependent pathway. In *Gas6*-silenced MII oocytes, spontaneous activation did not occur, and oocytes maintained long-term MII stage arrest (data not shown), suggesting that *Gas6* RNAi may not affect CSF activity.

Moreover, we observed that meiosis was completed with normal meiotic spindle organization and chromosome segregation in *Gas6*-silenced MII oocytes. The results indicate that *Gas6* is crucial for cytoplasmic maturation excluding cytoskeletal systems. In this study, *Gas6*-silenced mouse oocytes could complete nuclear maturation with decreased MPF activity near the time of the first polar body extrusion, and MPF activity remained low thereafter. We confirmed that these *Gas6*-silenced oocytes had abnormal oscillation patterns of intracellular Ca^2+^, no cortical granule exocytosis, and failure of fertilization due to the lack of PN formation. This indicated that nuclear and cytoplasmic maturation are uncoupled in *Gas6*-silenced oocytes, but both nuclear and cytoplasmic maturation are necessary to achieve complete developmental competence for fertilization. Cytoplasmic maturational events are required to initiate sperm head decondensation, DNA synthesis, and PN formation [53], [54].

After fertilization, oocytes undergo an increase in intracellular Ca^2+^ excitability, cortical granule exocytosis, and meiotic resumption with concurrent MPF inactivation and PN formation. Mammalian oocytes were arrested at metaphase of the second meiotic division. After sperm penetration, entailing sperm release sperm-specific phospholipase C to activate the phosphatidylinositol bisphosphate cascade, resulting in the production of inositol triphosphate (IP3) and diacylglycerol (DAG) [55]. IP3 stimulates the endoplasmic reticulum to release stored Ca^2+^ and facilitate oscillation [56]. Rescovitine, a specific inhibitor of p34cdc2/Cyclin B kinase inhibitor, was used to inactivate MPF in mouse oocytes, and suppressed fertilization-induced Ca^2+^ oscillations in normal MII eggs after first 1∼2 Ca^2+^ spikes. Therefore, inhibition of MPF activity in MII oocytes inhibits sperm-induced Ca^2+^ oscillations, suggesting that MPF plays an important role in regulation of the cytoplasmic Ca^2+^ excitability in mouse oocytes [57]. In this study, the low levels of MPF activity by *Gas6*-silencing and may be the low level of *Gas6* expression itself may alter the pattern of Sr^2+^-induced Ca^2+^ oscillations due to insufficient cytoplasmic maturation.

The pattern of repetitive Ca^2+^ oscillations in mice egg is essential for both early events of egg activation, such as the resumption of meiosis, cortical granules release, recruitment of maternal mRNA, and the subsequent normal developmental program [58], [59], [60]. After egg activation, concentrations of DAG and Ca^2+^ can activate protein kinase C (PKC). In conjunction with PKC, Ca^2+^ induces cortical granule exocytosis and the blockage of polyspermy [61]. When the calcium chelator BAPTA-AM was applied, no cortical granule exocytosis and no completion of meiosis occurred [62]. However, when a Ca^2+^ ionophore was applied, MII oocytes underwent cortical granule exocytosis, second polar body emission, and PN formation [63]. In this study, we observed that *Gas6-*silenced oocytes exhibited apparently low cytoplasmic Ca^2+^ excitability, followed by reduced cortical granule exocytosis and resulting in the failure of fertilization.

Initial sperm processing entails disassembly of the sperm nuclear envelop, which releases sperm components into the cytoplasm of oocytes, and this process is dependent upon oocyte-derived PKC [20]. Oocyte-derived glutathione and MPF activity are required for sperm decondensation through the reduction of protamine disulfide bonds [22], [64]. Subsequently, maternally supplied histones from oocyte cytoplasm are needed to recondense paternal chromatin via the assistance of various protein kinases and phosphatases [21], [23]. Previous research indicated that paternal chromatin remodeling is dependent upon the maturity of oocyte cytoplasm [65]. Indeed, Ca^2+^ oscillations promote histone assembly onto paternal chromatin [23]. In the present study, *Gas6*-silenced oocytes exhibited sperm penetration but no PN formation in the cytoplasm (Fig. 6). Due to the insufficient cytoplasmic maturation of oocytes after *Gas6* RNAi treatment, *Gas6*-silenced MII oocytes exhibited decreased MPF activity and concurrent cytoplasmic changes that require further study to reveal in the effects on paternal and maternal chromatin remodeling. In addition, *Gas6*-silenced, but MPF activity-rescued MII oocytes failed to undergo PN formation despite sperm penetration into the cytoplasm (Fig. 10A, B). These results suggest that the reduction of *Gas6* expression itself sufficient to affect cytoplasm maturation to induce failure of PN formation.

**Figure 11:**
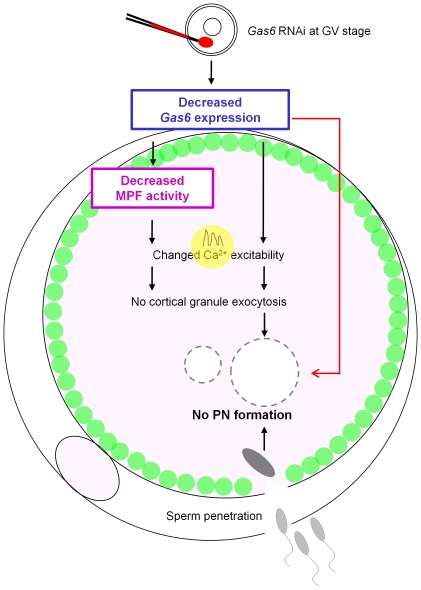
*Gas6* RNAi affects MPF activity and PN formation. When *Gas6* was silenced, oocytes developed to the MII stage with low MPF activity. These MII oocytes exhibited altered Ca^2+^ excitability and markedly reduced cortical granule exocytosis. In addition to these two well-known cytoplasmic changes related to the decreased MPF activity (black lines), novel mechanisms caused by *Gas6* knockdown (red line) may induce further aberrant changes of cytoplasmic maturation that permits sperm penetration but not PN formation. Gray circles with dotted lines designate failure of male and female pronuclei production. Green vesicles around the ooplasma membrane designate remained intact cortical granules.

In conclusion, this is the first report of the expression and function of *Gas6* in the nuclear and cytoplasmic maturation of oocytes and further process of fertilization. We propose *Gas6* as a new candidate mammalian maternal effect gene that is pivotal for oocyte cytoplasmic maturation and normal fertilization, especially for the low cytoplasmic Ca^2+^ excitability, and a lack of cortical granule exocytosis, paternal chromosome decondensation, and PN formation (Fig. 11).

## Materials and Methods

### Animals

ICR mice were obtained from Koatech (Pyeoungtack, Korea) and mated to male mice of the same strain to produce embryos in the breeding facility at the CHA Stem Cell Institute of Pochon CHA University. All described procedures were reviewed and approved by the University of Science Institutional Animal Care and Use Committee and performed in accordance with the Guiding Principles for the Care and Use of Laboratory Animals.

### Reagents

Chemicals and reagents were obtained from Sigma Chemical Co. (St. Louis, MO), unless noted otherwise.

### Isolation of oocytes and embryos

To isolate GV oocytes from preovulatory follicles, 4-week-old female ICR mice were injected with 5 IU of eCG and sacrificed after 46 hours later. Cumulus-enclosed oocyte complexes (COCs) were recovered from ovaries by puncturing the preovulatory follicles with 27-gauge needles. M2 medium containing 0.2 mM IBMX was used to inhibit GVBD. Isolated oocytes were snap-frozen and stored at −70°C prior to RNA isolation.

To obtain MII oocytes, we injected female mice with 5 IU eCG, followed by 5 IU hCG after 46 hours. Superovulated MII oocytes were obtained from the oviduct 16 hours after hCG injection. Cumulus cells surrounding MII oocytes were removed by treating COCs with hyaluronidase (300 U/ml). Female mice were superovulated and mated, and embryos were obtained at specific time points after hCG injection as follows: PN 1-cell embryo at 18–20 hours, 2C embryos at 44–46 hours, 4C embryos at 56–58 hours, 8C embryos at 68–70 hours, morula stage at 80–85 hours, and blastocyst stage at 96–98 hours.

### Messenger RNA isolation

Messenger RNA was isolated from oocytes and embryos by using the Dynabeads mRNA DIRECT kit (Invitrogen Dynal AS, Oslo, Norway) according to the manufacturer's instructions. To evaluate recovery, 0.1 ng of *Green Fluorescent Protein* (*GFP*) synthetic RNA were added to each oocyte or embryo prior to mRNA extraction [12]. Briefly, oocytes were resuspended in 300 µl of lysis/binding buffer (100 mM Tris-HCl [pH 7.5], 500 mM LiCl, 10 mM EDTA, 1% LiDS, 5 mM DTT) for 5 minutes at room temperature. After vortexing, 20 µl of prewashed Dynabeads oligo dT_25_ were mixed with lysate and annealed by rotating for 5 minutes at room temperature. The beads were separated with a Dynal MPC-S magnetic particle concentrator, and poly (A)^+^ RNAs were eluted by incubation in 10 µl of Tris-HCl (10 mM Tris-HCl, pH 7.5) at 65°C for 2 minutes.

### Reverse transcriptase (RT)-PCR

Complementary DNA was synthesized from mRNA or total RNA by using 0.5 µg of oligo dT primer according to the SuperScript Preamplification System protocol (Gibco-BRL, Grand Island, NY). PCR reactions (20 µl) contained 20 mM Tris-HCl (pH 8.4), 50 mM KCl, 1.5 mM MgCl_2_, 0.2 mM dNTPs, 25 pM of each primer, and 2.5 U of Taq DNA polymerase (Promega, Madison, WI). Single oocyte- and single embryo-equivalent cDNAs were used as templates for PCR analysis. PCR reaction conditions and primer sequences for the encoding genes are listed in Table 4. Set-A primers were used for dsRNA preparation, whereas Set-B was used for analyzing RNAi-mediated gene-specific knockdown by RT-PCR. Relative gene expression levels were normalized to the expression of *Gapdh*. All experiments were repeated in triplicate.

**Table 4:** Primer sequences and RT-PCR conditions used for *Gas6* RNAi.

Gene	Accession numbers	Primer sequence *	Annealing temperature	Product size
*Gas6*-A**	BC005444	For- CCGTGATTAGACTACGCTTCRev- AGTTGAGCCTGTAGGTAGCA	60°C	561 bp
*Gas6*-B**	BC005444	For- AAAGGGCCAGAGTGAAGTGARev- TTTTCCCGTTTACCTCCAGA	60°C	175 bp
*Gdf9*	NM_008110	For- GGTTCTATCTGATAGGCGAGGRev- GGGGCTGAAGGAGGGAGG	65°C	446 bp
*Plat*	NM_008872	For- CATGGGCAAGAGTTACACAGRev- CAGAGAAGAATGGAGACGAT	60°C	631 bp
*Mos*	NM_020021	For- TGGCTGTTCCTACTCATTTCRev- CTTTATACACCGAGCCAAAC	60°C	273 bp
*H1foo*	NM_138311	For- GCGAAACCGAAAGAGGTCAGAARev- TGGAGGAGGTCTTGGGAAGTAA	60°C	378 bp
*GFP*	EU056363	For- ATGGTGAGCAAGGGCGAGRev- CTTGTACAGCTCGTCCAT	60°C	717 bp
*Gapdh*	BC092294	For- ACCACAGTCCATGCCATCACRev- TCCACCACCCTGTTGCTGTA	60°C	451 bp

*For  =  Forward; Rev  =  Reverse.

**Primer set-A was used for the preparation of dsRNA, whereas set-B was used to confirm the gene-specific knockdown after RNAi.

### Western blot

Protein extract (500 oocytes per lane) was separated using 10% SDS-PAGE and transferred onto a polyvinylidene difluoride membrane (Amersham Biosciences, Piscataway, NJ). The membrane was blocked for 1 hour in Tris-buffered saline-Tween [TBST; 0.2 M NaCl, 0.1% Tween-20, and 10 mM Tris (pH 7.4)] containing 5% non-fat dry milk. The blocked membranes were then incubated with goat polyclonal anti-GAS6 antibody (1∶1000) or mouse monoclonal anti-α-tubulin antibody (1∶1000; sc-8035, Santa Cruz Biotechnology) in TBST. After incubation, membranes were incubated with horseradish-peroxidase-conjugated anti-goat IgG (1∶2000; A5420) or anti-mouse IgG (1∶2000; A-2554) in TBST for 1 hour at room temperature. After each step, the membranes were washed several times with TBST, and bound antibody was detected using an enhanced chemiluminescence detection system (Santa Cruz Biotechnology) according to the manufacturer's instructions.

### Preparation of *Gas6* dsRNA

For RNAi experiments, we prepared dsRNA for *Gas6* (561 bp). Two different primer sets, Set-A and Set-B, were used for dsRNA preparation and confirmation of knockdown of endogenous transcripts, respectively (Table 4). PCR for *Gas6* was performed using Set-A primers and resulted in a 561-bp product. Each *Gas6* cDNA was cloned into pGEM®-T Easy (Promega) and linearized with Spe I. Insert orientation was confirmed by PCR amplification by using the T7 primer with each Set-A primer. Single stranded RNA (ssRNA) for each orientation was synthesized using the MEGAscript RNAi Kit (Ambion, Austin, TX) and T7 RNA polymerase. Complementary RNAs were mixed and incubated for annealing at 75°C for 5 minutes, then cooled to room temperature. Formation of dsRNA was confirmed by 1% agarose gel electrophoresis, in which the mobility of dsRNA was compared to that of ssRNA. For microinjection, RNAs were diluted to a final concentration of 2 µg/µl.

### Microinjection and in vitro culture

GV oocytes were microinjected with *Gas6* dsRNA in M2 medium containing 0.2 mM IBMX. An injection pipette containing dsRNA solution was inserted into the cytoplasm of an oocyte, and 10 pl of dsRNA were microinjected using a constant flow system (Transjector; Eppendorf, Hamburg, Germany). To assess injection damage, oocytes were injected with elution buffer alone and used as sham controls. To determine the rate of in vitro maturation, oocytes were cultured in M16 medium containing 0.2 mM IBMX for 8 hours, followed by culture in M16 alone for 16 hours in 5% CO_2_ at 37°C. After RNAi experiments, in vitro maturation rates and morphological changes were recorded as previously described [12]. Briefly, the maturation stage of oocytes was scored by the presence of a germinal vesicle (GV oocyte), a polar body (MII oocyte), or neither a germinal vesicle nor a polar body (MI oocyte).

To confirm the effect of *Gas6*, oocytes were cultured in M16 medium containing 0.2 mM IBMX for 8 hours, followed by culture in M16 alone for 10 hours. After emission of first polar body, control oocytes were cultured in M16 containing 100 mM roscovitine or 1 mg/ml colcemid, while *Gas6*-silenced MII stage oocytes were cultured in M16 containing 1 mg/ml colcemid for 6 hours in 5% CO_2_ at 37°C. Roscovitine, a purine derivative, inhibit specifically the activity MPF [66], while colcemid, a well known microtubule-depolymerizing drug, maintain MPF activity by preventing the degradation of cyclin B in the mouse oocytes [67].

### Parthenogenetic activation and culture of activated oocytes

Mature oocytes were activated parthenogenetically by culturing for 2 hours in Ca^2+^-free KSOM medium supplemented with 10 mM SrCl_2_ and 5 mg/ml cytochalasin B. Modified Chatot, Ziomek, and Bavister (CZB) medium was used for the culture of parthenogenetically activated oocytes. Activated oocytes were cultured in CZB medium at 37°C in an air atmosphere containing 5% CO_2_ to monitor the development of activated oocytes to the 2C stage.

### Noninvasive examination of spindle structure

Spindle structure observation of living cells was performed using the LC Polscope optics and controller system combined with a computerized image analysis system (Oosight™ Meta Imaging System, CRI Inc., MA).

### Aceto-orcein staining

Oocytes were fixed with aceto-methanol (acetic acid:methanol = 1∶3) solution for 6 hours at 4°C. Fixed oocytes were transferred onto a slide and covered with a clean cover glass. Aceto-orcein solution was inserted between the slide and the cover glass, and the oocytes were photographed.

### Immunofluorescence staining

Denuded oocytes were placed in Dulbecco's PBS containing 0.1% polyvinyl alcohol (PBS-PVA), 4% paraformaldehyde, and 0.2% Triton X-100 and then fixed for 40 minutes at room temperature. Fixed oocytes were washed three times in PBS-PVA for 10 minutes each and stored overnight in 1% BSA-supplemented PBS-PVA (BSA-PBS-PVA). Oocytes were blocked with 3% BSA-PBS-PVA for 1 hour and incubated with mouse monoclonal anti-α-tubulin antibody (1∶100 dilution, sc-8035; Santa Cruz Biotechnology) at 4°C overnight. After washing, oocytes were incubated with FITC-conjugated anti-mouse IgG (1∶40) for 1 hours at room temperature, and DNA was counterstained with propidium iodide.

### Dual kinase activity assay

Oocytes were washed in 0.1% PBS-PVA, and then each oocyte was placed in an Eppendorf tube with 1 µl 0.1% PBS-PVA and 4 µl ice-cold extraction buffer (80 mM β-glycerophosphate, 25 mM HEPES [pH 7.2], 20 mM EGTA, 15 mM MgCl_2_, 1 mM DTT, 1 mM APMSF, 0.1 mM Na_3_VO_4_, 1 µg/ml leupeptin, and 1 µg/ml aprotinin). Samples were frozen at −80°C until the assay. After thawing, oocytes were centrifuged at 13,000× *g* for 3 minutes, followed by the addition of 5 µl kinase buffer and 5 µl substrates, and incubation for 20 minutes at 37°C. The kinase buffer comprised 75 mM HEPES (pH 7.2), 75 mM β-glycerophosphate, 75 mM MgCl_2_, 6 mM DTT, 10 mM EGTA, 60 µM ATP, 15 µM cAMP-dependent protein kinase inhibitor peptide and 0.3 µCi/µl [γ-^32^P]-ATP (250 µCi/25 µl; Amersham Pharmacia Biotech). The substrate solution for the MPF and MAPK double kinase assay contained 4.5 µl histone H1 (5 mg/ml, calf thymus) and 0.5 µl myelin basic protein (MBP; 5 mg/ml, bovine brain). The reaction was terminated by the addition of 5 µl 4× SDS sample buffer and boiling for 5 minutes. Samples were separated by 15% PAGE, and labeled MBP and histone H1 were analyzed by autoradiogram.

### In vitro fertilization

Sperm were collected from the cauda epididymis of 8-week-old male ICR mice (Koatech). The tissues were incised, and the sperm were allowed to swim out into M16 medium. Sperm were incubated in M16 medium for 1 hour to allow capacitation. The ZP was removed from oocytes by dissolution in Tyrode's solution (pH 2.5). When ZP thinning was observed through the microscope, oocytes were transferred to M16 medium, and the cellular mass was washed out of the ZP by gentle pipetting. ZP-free MII eggs, which had been placed previously in a 200-µl M16 medium droplet under mineral oil, were inseminated with 2.5×10^4^/ml sperm for 2 hours, and then oocytes were washed free of unbound sperm. Washing and transfer of the oocyte were necessary to avoid excessive polyspermy of ZP-free oocytes. The oocytes were transferred into M16 medium for culture in 5% CO_2_ at 37°C for 5 hours to observe PN formation.

### Measurement of intracellular Ca^2+^ concentration

Changes in the intercellular calcium concentration, [Ca^2+^]_i_, were measured by loading oocytes with 1 µM of the Ca^2+^-sensitive indicator dye Fluo-4AM (Molecular Probes, Eugene, OR) supplemented with 0.02% pluronic acid (Molecular Probes) in HEPES-buffered Tyrode's lactate solution. Changes in [Ca^2+^]_i_ were monitored using an Axiovert 200M microscope fitted with a 10× objective lens and CCD camera controlled by Axiovision software 4.8.1 (Carl Zeiss, Jena, Germany). Intracellular calcium concentrations were monitored by measuring fluorescence from individual oocytes loaded on a temperature-controlled chamber dish (SEC, Seoul, Korea) coated with CellTak® (BD Biosciences, San Jose, CA). Changes in the fluorescence intensities of [Ca^2+^]_i_ were obtained every 20 seconds.

### Observation of exocytosis of cortical granules

Oocytes were labeled with lectins according to the previously reported method [68]. Immediately after removal of the ZP, oocytes were fixed in 3% paraformaldehyde in PBS for 30 minutes at 20°C. After fixation, oocytes were rinsed with blocking solution, PBS containing 1% BSA, and 100 mM glycine, for 10 minutes to remove aldehyde. Oocytes were permeabilized with 0.1% Triton X-100 in PBS for 5 minutes. After washing twice in blocking solution, oocytes were incubated with solution of 200 µg/ml FITC-conjugated LCA lectin for 30 minutes. Oocytes were rinsed thoroughly with PBS and transferred to PBS-PVA. Chromatin condensation was visualized by costaining with 10 µg/ml 4,6-diamidino-2-phenyl indole for 10 minutes. Oocytes were mounted between a slide and coverslip.

### Statistical analysis

Statistical analyses of real-time PCR data were evaluated using one-way analysis of variance and a log linear model. Data were presented as mean ± SEM, derived from at least 3–5 separate and independent experiments, and a value of *p*<0.05 was considered statistically significant.
